# Whole Exome Sequencing Uncovers Germline Variants of Cancer-Related Genes in Sporadic Pheochromocytoma

**DOI:** 10.1155/2018/6582014

**Published:** 2018-08-19

**Authors:** Milena Urbini, Margherita Nannini, Annalisa Astolfi, Valentina Indio, Valentina Vicennati, Matilde De Luca, Giuseppe Tarantino, Federica Corso, Maristella Saponara, Lidia Gatto, Donatella Santini, Guido Di Dalmazi, Uberto Pagotto, Renato Pasquali, Andrea Pession, Guido Biasco, Maria A. Pantaleo

**Affiliations:** ^1^“Giorgio Prodi” Cancer Research Center, University of Bologna, Bologna, Italy; ^2^Department of Specialized, Experimental and Diagnostic Medicine, S. Orsola-Malpighi Hospital, University of Bologna, Bologna, Italy; ^3^Endocrinology Unit, Department of Medical and Surgical Sciences, Center for Applied Biomedical Research, S. Orsola-Malpighi Hospital, University of Bologna, Bologna, Italy; ^4^Pathology Unit, S. Orsola-Malpighi Hospital, University of Bologna, Bologna, Italy

## Abstract

**Background:**

Pheochromocytomas (PCCs) show the highest degree of heritability in human neoplasms. However, despite the wide number of alterations until now reported in PCCs, it is likely that other susceptibility genes remain still unknown, especially for those PCCs not clearly syndromic.

**Methods:**

Whole exome sequencing of tumor DNA was performed on a set of twelve PCCs clinically defined as sporadic.

**Results:**

About 50% of PCCs examined had somatic mutations on the known susceptibility *VHL*, *NF1*, and *RET* genes. In addition to these driver events, mutations on *SYNE1*, *ABCC10*, and *RAD54B* genes were also detected. Moreover, extremely rare germline variants were present in half of the sporadic PCC samples analyzed, in particular variants of *MAX* and *SAMD9L* were detected in the germline of cases wild-type for mutations in the known susceptibility genes.

**Conclusions:**

Additional somatic passenger mutations can be associated with known susceptibility *VHL*, *NF1*, and *RET* genes in PCCs, and a wide number of germline variants with still unknown clinical significance can be detected in these patients. Therefore, many efforts should be aimed to better define the pathogenetic role of all these germline variants for discovering novel potential therapeutic targets for this disease still orphan of effective treatments.

## 1. Introduction

Pheochromocytomas (PCCs) are rare tumors of the autonomic nervous system that arise from the chromaffin tissue of the adrenal medulla [1]. Most of PCCs are benign; however, approximately 10% of cases are malignant and can develop metastases either at the time of diagnosis or even later after several years, with a highly variable clinical course and a 5-year overall survival rate of 50% [2]. PCCs show the highest degree of heritability in human neoplasms and almost 40% of cases occur within heritable syndromes, including multiple endocrine neoplasia type 2 (MEN2), neurofibromatosis type 1 (NF1), von Hippel Lindau (VHL) disease, and hereditary paraganglioma and familial pheochromocytoma [3]. Germline mutations have been identified in more than 15 well-characterized genes, such as *VHL*, *SDHB*, *SDHD*, *NF1*, and *RET* [4].

Despite this well-known inherited basis of PCCs and paragangliomas (PGLs), during the past decades somatic mutations with variable frequency in many genes, including *EPAS1* (*HIF2α*), *RET*, *VHL*, *RAS*, *NF1*, *ATRX*, and CSDE1 recurrent somatic copy number alterations and several fusion genes, involving *MAML3*, *BRAF*, *NGFR*, and *NF1*, have been progressively identified [5–16]. Recently a multiplatform integrated analysis classified PCCs/PGLs into four clinically relevant molecular subtypes: a kinase signaling subtype, a pseudohypoxia subtype, a Wnt-altered subtype driven by *MAML3* and *CSDE1*, and a cortical admixture subtype [16].

Therefore, given this molecular complexity of PCCs, it is likely that other alterations remain still unknown, especially for those PCCs not clearly syndromic. For this purpose, in the present study, we performed whole exome sequencing on a set of 12 clinically sporadic PCCs, with a family history negative for PCCs/PGLs.

## 2. Materials and Methods

### 2.1. Patients and Tumor Samples

Fresh tissue specimens of PCC from 12 patients with a family history negative for PCCs/PGLs were collected during the surgical operation, snap-frozen in liquid nitrogen, and stored at −80°C until analysis. Patient characteristics are listed in Table 1. Whole exome sequencing was performed on biological tumor samples on matched peripheral blood samples obtained from all patients. This study was approved by the local institutional ethical committee of S. Orsola-Malpighi hospital (approval number 95/2013/U/Tess). All patients provided written informed consent.

### 2.2. Whole Exome Sequencing

DNA was extracted from peripheral blood and fresh frozen tissue with DNA mini kit (Qiagen, Milan, Italy) following manufacturer's instructions. Whole exome sequencing of tumor DNA was performed on HiScanSQ platform in accordance with Nextera Rapid Exome Enrichment protocol (Illumina, San Diego, California, USA). Briefly, 100 ng of genomic DNA was tagged and fragmented by the Nextera transposome. The Nextera transposome simultaneously fragments the genomic DNA and adds adapter sequences to the ends. The products were then amplified and exome regions were enriched. The enriched libraries were amplified by PCR and quantified using PicoGreen assay (Life Technologies, Milan, Italy).

Paired-end libraries were sequenced at 2 × 100 bp read length using Illumina Sequencing by synthesis (SBS) technology.

### 2.3. Bioinformatic Analysis

After demultiplexing and FASTQ generation performed with bcltofastq function developed by Illumina, the paired-end reads were trimmed using AdapterRemoval (https://github.com/MikkelSchubert/adapterremoval) with the aim of removing stretches of low-quality bases (<Q10) and Truseq/Nextera rapid capture adapters present in the sequences. The paired-end reads were then aligned on human reference genome hg38 (http://www.genome.ucsc.edu). Data from WES were mapped with Burrows-Wheeler Aligner with the default setting; the PCR and optical duplicates were removed, and Genome Analysis Toolkit (https://software.broadinstitute.org/gatk) was used to locally realign, recalibrate, and call the Ins/del variants, while point mutations were identified with the algorithm MuTect (https://www.broadinstitute.org/cancer/cga/mutect). Single nucleotide variants (SNV) and ins/del were annotated with gene and protein alteration using Annovar (http://annovar.openbioinformatics.org); nonsynonymous and nonsense SNV, frameshift/nonframeshift Indels, and splice site mutations were selected with a threshold read depth ≥ 10x and a variant allele frequency ≥ 0.2. All the variants were filtered to select novel or rare events basing on database of human variability dbSNP (http://www.ncbi.nlm.nih.gov/SNP), 1000Genomes (http://www.1000genomes.org), ExAC (http://exac.broadinstitute.org), and EVS (http://evs.gs.washington.edu/EVS). In-depth evaluation of high confidence somatic variants was performed by verifying the presence of alternate allele on the normal counterpart and manually visualizing each variation with the *tview* function of SAMmtools. Both somatic mutations and germline variants were searched in COSMIC (Catalog of Somatic Mutations in Cancer; http://cancer.sanger.ac.uk/cosmic), ClinVar (https://www.ncbi.nlm.nih.gov/clinvar), and HGMD (http://www.hgmd.org), and their effect on protein structure and function was predicted with SNPeff, a software that uses three different prediction algorithms (SIFT, Polyphen2, and LRT).

Moreover, based on WES data, the analysis of amplifications and large deletions were performed making a consensus between Control FREEC (http://boevalab.com/FREEC) and ADTEX (http://adtex.sourceforge.net) with paired tumour/matched normal samples. A filtering procedure was applied taking into account the uncertainty value given by Control FREEC (<80%) and the polymorphic copy number variants from the Database of human Genomic Variants (http://dgv.tcag.ca/dgv/app/home).

For germline variants prioritization, all rare (MAF < 0.01) alterations occurring on the known susceptibility genes of PCC and PGL were considered. Moreover, variants with an evident effect on the protein (nonsense and splicing mutations or frameshift ins/del) were prioritized and manually annotated using HGMD and ClinVar database and with literature.

### 2.4. Sanger Sequencing

Sequencing of the DNA extracted from tumors and matched peripheral blood samples was performed to validate candidate mutations. Specific PCR assay for the amplification and sequencing of selected genes was designed with Primer Express 3.0 Software (Applied Biosystems, Monza, Italy). PCR products were purified with the Qiaquick PCR purification kit (Qiagen) and sequenced on both strands using the Big Dye Terminator v1.1 Cycle Sequencing kit (Applied Biosystems). Sanger Sequencing was performed on ABI 3730 Genetic Analyzer (Applied Biosystems).

### 2.5. Real-Time PCR

Total RNA was extracted from fresh frozen tissues using the RNeasy spin-column method (Qiagen). RNA was reverse transcribed to cDNA using the Transcriptor First-Strand cDNA Synthesis Kit (Life Technologies) with oligo dT primers. qPCR amplification of genes of interest was performed with real-time LightCycler 480 instrument (Roche). Fold-change was estimated by DDCt method, using ATPS, HPRT, and HMBS genes as housekeeping controls. Primers used were: MAX_FW 5′- GCGATAACGATGACATCGAGGT-3′ and MAX_RV 5′-CCCGCAAACTGTGAAAGCTGT-3′, SAMD9L_FW 5′-AAAGTGAGTGAGTGAGCCCAG-3′ and SAMD9L_RV 5′-CATGCTCTTTGGTCCAGTCT-3′, ATPS_FW 5′-GTCTTCACAGGTCATATGGGGA-3′ and ATPS_RV 5′-ATGGGTCCCACCATATAGAAGG-3′, HMBS_FW 5′-GGCAATGCGGCTGCAA-3′ and HMBS_RV 5′-GGGTACCCACGGAATCAC-3′, HPRT_FW 5′-TGACACTGGCAAAACAATGCA-3′ and HPRT_RV 5′-GGTCCTTTTCACCAGCAAGCT-3′. For detection of cortical admixture profile, expression levels of *STAR*, *CYP2W1*, *CYP11B2*, *CYP21A2*, and *CLND2*, genes were evaluated using the following primers: STAR_Fw 5′-TGGGCATCCTTAGCAACCAA-3′ and STAR_Rev 5′-GCCCACATCTGGGACCACTT-3′; CYP2W1_Fw 5′-GTCATGGTCCTCTTGGGGTC-3′ and CYP2W1_Rev 5′-CTCCAGGAGGGTCCTCAGAA-3′; CYP11B2_Fw 5′-TGCATCCCTGCAGGATGAT-3′ and CYP11B2_Rev 5′-GCGACAGCACATCTGGGT-3′; CYP21A2_Fw 5′-AGCCCGACCTCCCCAT-3′ and CYP21A2_Rev 5′-CACCACCACATCTTGCAGCC-3′; CLND2_Fw 5′-CCCCTTGTACTTCGCTCCCC-3′ and CLND2_Rev 5′-AAGCAGCCTCAAGAAGGCATC-3′.

## 3. Results

Exome sequencing generated a minimum of 45.5 million reads/sample with a mean coverage of RefSeq regions of 44x. Few somatic mutations were identified (an average of 7 mutations per sample) while several copy number alterations were detected, with losses of chr1, chr3, and chr17 being the most recurrent.

### 3.1. Analysis of Somatic Mutations

**Figure 1:**
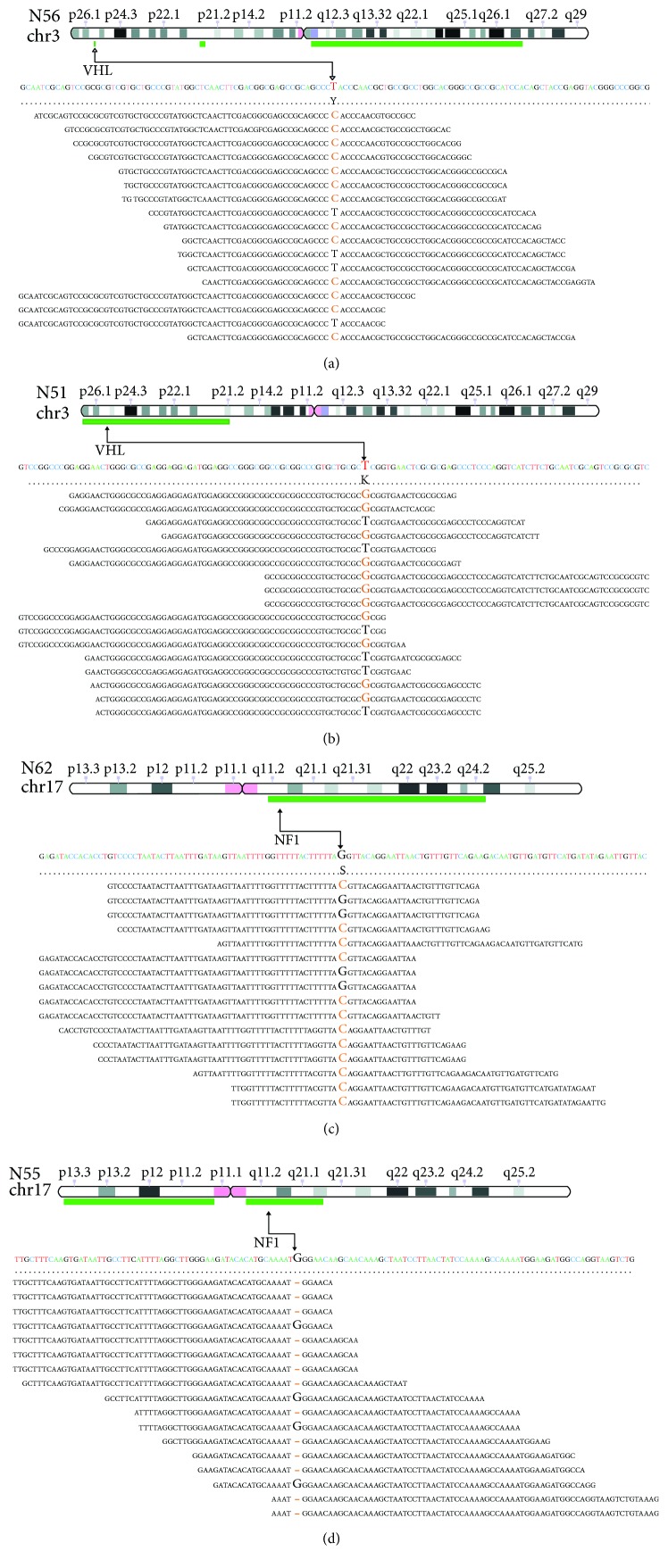
Deletion of the wild-type allele occurred in PCC samples that carry *NF1* or *VHL* somatic mutations. Alignments of sequencing reads located on *NF1* or *VHL* mutated bases are shown. Green bars represent deleted regions of chr3 for *VHL* (a, b) and chr17 for *NF1* (c, d) detected by copy number analysis. A black arrow indicates the chromosome position of *NF1* or *VHL* and the mutated base on the sequencing reads.

The biological effect of somatic mutations was predicted with three bioinformatic tools (Suppl.  Table 1). Mutations on susceptibility genes were detected in 6 out of 12 cases and were annotated in COSMIC and HGMD databases. *VHL* missense mutations were identified in two cases: a p.S65A identified in N51, a mutation already reported in PCC (COSM144970), and a p.Y98H in N56, a mutation reported in ClinVar and HMGD as pathogenic in association with Von Hippel-Lindau syndrome. Two novel somatic alterations were detected in *NF1*: N62 carried a splicing mutation c.480-1G>C in exon 5 and N55 had a frameshift deletion (p.W784fs) in exon 20. Interestingly, the abovementioned mutations on *NF1* and *VHL* were in regions affected by loss of the wild-type allele (Figure 1). Heterozygous loss of the chromosomal region covering *NF1* was detected also in N54; however, no additional mutational event was detected on this gene. *RET* was found mutated in heterozygosis in two other tumors: a novel exon 11 nonframeshift INDEL (p.L633delinsLCR) was detected in N57 and the hotspot mutation p.M918T (COSM965) in N53.

In addition to these driver mutations on known susceptibility genes, other somatic passenger mutations were detected in these 6 tumors. In particular, *SYNE1* and *ABCC10* were mutated, respectively, in the two *RET*-mutated cases (N53 and N57), while a missense p.G460S mutation of *RAD54B*, a gene involved in DNA repair process, affected a NF1 mutated case (N55). On the other side, few somatic mutations were identified in the 6 remaining PCC cases and none was recurrent between samples. N63 carried a p.L114X nonsense mutation in *CDC14B*, a protein phosphatase involved in DNA damage response. N52 carried two heterozygous missense mutations (*SMARCC2* p.P1092R and *PRKG1* p.F387) both predicted as pathogenic by bioinformatic predictors.

### 3.2. Analysis of Germline Rare Variants

**Figure 2:**
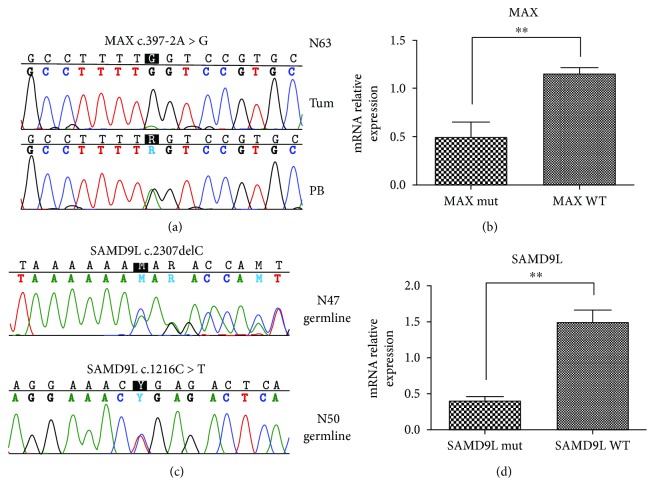
(a) Loss of heterozygosity of germline *MAX* mutation detected on N63 tumor. Chromatograms showing homozygous status of c.397-2A>G mutation in the tumor of N63 (upper panel), while in the germline it is heterozygous (lower panel). (b) *MAX* mRNA relative expression of *MAX* mutant case (N63) in comparison to the other PCC cases. *P* value was estimated with unpaired *t*-test (^∗∗^ *P* < 0.01). (c) Validation of SNV detected on *SAMD9L* gene in 2 PCC samples. Germline c.2307delC and c.1216C>T heterozygous mutations detected in both tumor and peripheral blood of N47 and N50, respectively. (d) *SAMD9L* mRNA relative expression of the 2 *SAMD9L* mutant cases (N47 and N50) in comparison to the other PCC cases. *P* value was estimated with unpaired *t*-test (^∗∗^ *P* < 0.01).

In addition to the abovementioned somatic alterations, rare (ExAc < 0.01) germline variants were identified in five sporadic PCC samples (Suppl.  Table 2), among which those occurring in *MAX* and *SAMD9L* seem to play an important role in PCC pathogenesis, completing the picture of relevant alterations identified in our cohort (Table 2). In N63, a novel germline variant of *MAX* (c.397-2A>G), affecting splice site and pathogenic for the protein function, was detected. Noticeably, this patient is a young adult (age 26) and showed the loss of the wild-type allele in the tumor, thus following the Knudson two-hit model (Figure 2(a)), and mRNA level of *MAX* was found downregulated with respect to the other PCC samples (Figure 2(b)). According to the findings of Fishbein et al. [16], we evaluated the expression level of *STAR*, *CYP11B2*, *CYP2W1*, *CYP21A2*, and *CLND2* with the aim to assess whether a cortical admixture profile was present in this *MAX* mutated sample. However, a general low expression level of these genes was found in our cohort and no difference between the *MAX* mutated case and the other PCC samples was detected (data not shown). Conversely, 2 germline variants of *SAMD9L* were found in other 2 PCC, both *wild-type* for mutations in known susceptibility genes: a novel p.N769fs frameshift deletion in N47 and a rare (ExAc = 0.2%) nonsense p.R406X in N50 (Figure 2(c)). Evaluation of mRNA level of *SAMD9L* showed a significant downregulation of the transcript in this two PCC samples (Figure 2(d)). Interestingly, the same gene was found somatically mutated in N54 (p.L1016S), increasing to 3 the number of PCC *wild-type* cases of our cohort that carried alterations on *SAMD9L* (Table 2).

On the contrary, the role of the remaining germline variants (suppl.  Table 2) is not clear. In N50, in addition to *SAMD9L* alteration, a heterozygous stop gain of *BRCA2* (p.K3326X) was detected as constitutive. This variant is recorded in ClinVar as “benign”; however, it is found at low frequency in healthy individuals (ExAc allele frequency = 0.7%). *BRCA2* mRNA expression level was evaluated, but it was found not altered in this case with respect to the other PCC cases (data not shown). Finally, the rare germline variants of *ATRX* and *KTM2D*, identified in N56, and of *MDH2*, in N53, are missense variants occurring in association with a well-defined somatic mutation on a susceptibility gene (*VHL* in N56 and *RET* in N53) and then it is not clear whether they could have a role on the tumor onset.

## 4. Discussion

In this study, we performed whole exome sequencing on a set of twelve PCCs, clinically defined as sporadic, and we found that 50% of PCCs examined had somatic mutations on the known susceptibility *VHL*, *NF1*, and *RET* genes. In addition to these driver mutations, other somatic passenger mutations were detected. In particular, *SYNE1* and *ABCC10* were mutated, respectively, in the two RET-mutated cases, while a missense p.G460S mutation of *RAD54B* affected a NF1 mutated case, suggesting these other events may play a potential role on PCCs pathogenesis and development. Nuclear envelope 1 (*SYNE1*) gene encodes several different isoforms involved in a variety of cellular processes including cytokinesis, Golgi function, and nuclear organization and structural integrity and positioning of the nucleus [17, 18]. Mutations of *SYNE1* have been found in colorectal cancer, glioblastoma, and ovarian cancer, and methylation of the gene was also frequently found in lung adenocarcinoma and colorectal cancer [19–23]. On the other side, *RAD54B* is a telomere-related gene involved in DNA repair process, and coding-missense changes of this gene have been found in familial breast cancer cases not explained by mutations in the best-known high susceptibility genes *BRCA1* and *BRCA2* [24]. These findings may be even more relevant in PCCs, given the highest degree of heritability of this disease.

Of note on this topic, the identification of germline variants in half of the sporadic PCC samples was analyzed, among which those occurring in *MAX* and *SAMD9L* genes may be extremely interesting. Indeed, some reports disclose the importance of screening for germline variants also in sporadic cases, especially on susceptibility genes [25, 26], and besides the potential role played in PCCs pathogenesis, the detection of these germline variants in patients clinically defined as sporadic may suggest the existence of unknown multineoplasia hereditary diseases.

In our study, we have identified a variant of *MAX* (c.397-2A>G), affecting splice site and pathogenic for the protein function, with the loss of the wild-type allele in the tumor as second hit. Of note, this patient is a young adult of 26 years old, and the presence of a germline variant in a known cancer susceptibility gene may suggest that the PCC in this patient could be the first clinical expression of a hereditary disease still undefined. It is already known that around 1% of PCC patients were negative for mutations in the other known susceptibility genes carried a germline mutation affecting *MAX* [26, 27]. About 20 variants affecting *MAX* have been already described distributed along the gene, but more frequently involving exons 3 and 4, matching some of the most important residues within the conserved bHLH-Zip domain of *MAX*. Most mutations lead to truncated proteins, with the expected LOH affecting the remaining wild-type allele of the *MAX* tumor suppressor gene [26, 27]. Two truncating *MAX* mutations affecting exon 3 (c.97C>T) and 4 (c.185_186delA) and three missense variants (c.67G>A, c.281T>C, and c.425C>T) located in exons 3, 4, and 5, respectively, have been identified [27]. Furthermore, other mutations affecting the initial methionine (c.2T>A), creating a premature stop codon (c.25del, c.97C>T, c.223C>T, and c.244C>T) or affecting a donor/acceptor splice site (c.171 + 1G>A and c.295 + 1G>T), have been subsequently reported [26]. In addition, 2 deletions were identified: the first caused an inframe loss of 6 highly conserved amino acids within the first helix of the protein (c.140_157del), and the second, spanned the whole gene (c.1-?_483+?del) [26]. The *MAX* mutant case of our cohort showed a marked downregulation of *MAX* at mRNA level; however, it did not show a significant modulation of the genes involved in cortical admixture phenotype. Conversely to this finding, two cases of *MAX* mutant PCC were described to overexpress adrenal cortex markers (including *CYP11B2*, *CYP21A2*, and *STAR*) supporting an association between *MAX* mutation and the cortical admixture PCC subtype [16]. Further studies on larger cohort will be needed to assess this association.

Moreover, we have found three germline variants in *BRCA2* and *SAMD9L* in other two PCC patients wild-type for mutations in susceptibility genes. Specifically, even if it is a rare variant (ExAc allele frequency = 0.7%) recently described as associated with an increased risk of developing breast and ovarian cancers [28], the heterozygous stop gain of *BRCA2* (p.K3326X) is considered as “benign” in ClinVar. In support of this consideration, we did not detect any variation of *BRCA2* mRNA expression level. Thus, we cannot draw any conclusion on the role of this variant and PCC onset. On the contrary, the 2 rare germline variants of *SAMD9L* detected in two sporadic PCC cases were both producing a premature STOP of the protein (a p.N769fs frameshift deletion and a nonsense p.R406X) and were associated with significant downregulation of *SAMD9L* mRNA, supporting the pathogenicity of these alterations. The function of this gene is not well characterized; however, evidence has accumulated supporting the role of *SAMD9L* in cell proliferation and tumor suppression. In particular, somatic mutations have been found in hepatitis B-related hepatocellular carcinomas [29] and inactivation of *SAMD9L* has been recently correlated with myeloid transformation [30]. Interestingly, a somatic p.L1016S mutation of SAMD9L was detected in another PCC sample of our cohort, and additionally a p. P636S (COSM3412478) was reported in one case of TCGA PCC/PGL dataset (http://cancergenome.nih.gov/). Taken together, these data could support a possible role of *SAMD9L* in PCC biology; however, functional studies will be needed to further assess this hypothesis.

## 5. Conclusions

Taken together, the discovery of novel germline variants of known cancer-related genes in sporadic PCC patients, wild-type for mutations in susceptibility genes, may suggest that the likely existence of other multineoplasia syndrome. On the contrary, the role of the other rare germline variants *ATRX*, *KTM2D*, and *MDH2* genes identified in two cases having a well-defined somatic mutation on a susceptibility gene (*RET* or *VHL*) remains still unclear.

Although the pathogenetic role of all these variants is still not known, due to the large number of susceptibility genes implicated in the diagnosis of inherited PCCs and PGLs, we confirm also what other authors stated that the next-generation sequencing technology is ideally suited for carrying out genetic screening of these individuals [31]. Moreover, the high degree of heritability of PCCs and the wide number of germline variants described suggest the likely need of a more extended genetic counselling and the type and duration of the surveillance program of patients affected by PCCs with variants of unknown significance. Finally, these findings may underlie the possible occurrence of novel hereditary diseases that remain still undefined.

In conclusion, additional somatic passenger mutations can be associated to known susceptibility *VHL*, *NF1*, and *RET* genes in PCCs and a wide number of germline variants with still unknown clinical significance can be detected in these patients. Therefore, many efforts should be aimed to better define the pathogenetic role of all these germline variants for discovering novel potential therapeutic targets for this disease still orphan of effective treatments.

## Figures and Tables

**Table 1:** Patient characteristics.

ID	Sex	Age	Tumor size (cm)	HIC characteristics	PASS score
N47	F	63	5.0	Ki-67 2.4%	n.a.
N49	M	65	4.0	Positive staining for synaptophysin. Ki-67 3.2%	3
N50	M	36	4.0	Positive staining for synaptophysin. S100 protein-positive sustentacular cells. Ki-67 2.2%	3
N51	M	46	1.5	Positive staining for chromogranin A and synaptophysin. S100 protein-positive sustentacular cells. Negative staining for c-kit, EGFr, p53. Ki-67 0.5%	2
N52	M	47	3.5	Positive staining for chromogranin A and synaptophysin. S100 protein-positive sustentacular cells. Negative staining for CD10. Ki-67 0.1%	4
N53	F	58	5.4	Ki-67 1.8%	5
N54	F	46	6.0	Positive staining for chromogranin A and synaptophysin. S100 protein-positive sustentacular cells. Focal positive staining for CD10. Ki-67 0.4%	6
N55	F	30	6.0	Positive staining for chromogranin A and synaptophysin. S100 protein-positive sustentacular cells. Ki-67 0.1%	4
N56	M	41	1.7	Positive staining for synaptophysin. S100 protein-positive sustentacular cells. Negative staining for calretinin. Ki-67 2.2%	2
N57	M	35	8.0	Positive staining for chromogranin A and synaptophysin. Few S100 protein-positive sustentacular cells. Negative staining for calretinin and *α*-inhibin. Ki-67 5.7%	4
N62	M	59	3.0	Ki-67 1.4%.	5
N63	M	26	7.0	Positive staining for synaptophysin. S100 protein-positive sustentacular cells. Negative staining for calretinin, MEL-A, and *α*-inhibin. Ki-67 4%	14

**Table 2:** Somatic and germline alterations identified in our cohort that could support PCC pathogenesis. Annotations concerning Exac frequency, COSMIC, ClinVar, and biological effect predictions are shown for each variant.

Sample	Gene	Somatic/germline	Position	Exon	cDna	Protein	Type	dbSNP	ExAC freq	COSMIC	ClinVar	Effect prediction^§^
N47	SAMD9L	Germline	chr7:93133665	5	c.2307delC	p.N769fs	Frameshift	Novel	—	Yes	—	Pathogenic
N50	SAMD9L	Germline	chr7:93134756	6	c.1216C>T	p.R406X	Stop gain	rs150070697	0.002	Yes^∗^	—	Pathogenic
N54	SAMD9L	Somatic	chr7:93132925	4	c.3047T>C	p.L1016S	Missense	Novel	na	—	—	Pathogenic
N63	MAX	Germline SNV + somatic loss	chr14:65076665	7	c.397-2A>G	na	Splicing	Novel	—	Yes	—	Pathogenic
N51	VHL	Somatic + loss	chr3:10142040	1	c.193T>G	p.S65A	Missense	Novel	—	Yes ^∗^	—	Pathogenic
N56	VHL	Somatic + loss	chr3:10142139	1	c.292T>C	p.Y98H	Missense	rs5030809	—	Yes	Pathogenic	Pathogenic
N55	NF1	Somatic + loss	chr17:31227548	20	c.2351delG	p.W784fs	Frameshift	Novel	—	Yes	—	Pathogenic
N62	NF1	Somatic + loss	chr17:31169890	5	c.480-1G>C	na	Splicing	Novel	—	Yes	—	Pathogenic
N53	RET	Somatic	chr10:43121968	16	c.2753T>C	p.M918T	Missense	rs74799832	—	Yes ^∗^	Pathogenic	Pathogenic
N57	RET	Somatic	chr10:43114499	11	c.1899_1900insTGCCGC	p.L633delinsLCR	Nonframeshift insertion	Novel	—	Yes ^∗^	—	Pathogenic

^∗^COSMIC record that totally match with the mutation identified; ^§^ defined pathogenic if the mutation has been classified as pathogenic or deleterious by at least two out three predictors used (SIFT, Polyphen2, and LRT).

## Data Availability

All sequencing data are available upon request to the corresponding author.

## Supplementary Materials

Supplementary Materials Supplementary Table 1: list of somatic mutations identified in our cohort. Annotations concerning ExAC frequencies, COSMIC, and effect prediction are shown for each variant. In yellow are highlighted mutations affecting PCC susceptibility genes. Supplementary Table 2: list of rare germline variants identified. Annotations concerning Exac frequency, COSMIC, ClinVar, and biological effect predictions (SIFT, Polyphen2, and LRT) are shown for each variant.
